# Kalmer, a specific based-App intervention for the treatment of Non-suicidal self-injury (NSSI): a technical and usability study in a non-clinical population

**DOI:** 10.3389/fdgth.2026.1794576

**Published:** 2026-06-03

**Authors:** Mónica Conesa Giménez, Irene Jaén, Daniel Vega, Anna Julià, Marina López-Solà, Jordi Solé-Casals, Ignacio Miralles, Azucena Garcia-Palacios

**Affiliations:** 1 Department of Basic and Clinical Psychology and Psychobiology, Jaume I University, Castellon, España; 2 Department of Developmental, Educational, Social, and Methodology Psychology, Jaume I University, Castellon, España; 3 Consorci Sanitari de L'Anoia I Fundació Sanitària d’Igualada, Hospital d’Igualada, Igualada, Spain; 4 Department of Psychiatry and Forensic Medicine, Universitat Autònoma de Barcelona, Barcelona, Spain; 5 Institute of Neurosciences, University of Barcelona, Barcelona, Spain; 6 Serra Hunter Programme, Department of Medicine, School of Medicine and Health Sciences, University of Barcelona, Barcelona, Spain; 7 Institut d’Investigacions Biomèdiques August Pi i Sunyer (IDIBAPS), Barcelona, Spain; 8 Data and Signal Processing Research Group, University of Vic-Central, University of Catalonia, Vic, Catalonia, Spain; 9 Department of Psychiatry, University of Cambridge, Cambridge, United Kingdom; 10 Institute of New Imaging Technologies, Jaume I University, Castellon, España; 11 CIBERObn Physiopathology of Obesity and Nutrition, Instituto de Salud Carlos III, Madrid, Spain

**Keywords:** adolescents and young people, ecological momentary intervention, m-Health technology, mobile computing, non-suicidal self-injury, psychology, user experience

## Abstract

**Introduction:**

Non-suicidal self-injury (NSSI), defined as the deliberate infliction of harm to oneself without suicidal intent, poses a significant and growing mental health concern worldwide, particularly among adolescents and young adults. This behavior is associated with considerable emotional, social, and economic burdens, and is closely linked to heightened risks of future psychological challenges, including suicidal behavior. Digital interventions based on Ecological Momentary Assessment (EMA) and Ecological Momentary Intervention (EMI) offer promising solutions by overcoming access barriers of traditional therapies. This study introduces Kalmer, a mobile app designed to aid young individuals (ages 14–25) engaging in NSSI, integrating Cognitive Behavioral Therapy (CBT) and Dialectical Behavior Therapy (DBT) strategies.

**Methods:**

A usability study was conducted with 26 non-clinical participants aged 16–25, evaluating app's functionality, satisfaction, and acceptability.

**Results:**

Results indicate high usability scores (SUS mean: 81) and positive perceptions regarding quality. Users assess the objective quality to be good (mean = 3.88/5), subjective quality to be improvable (mean = 2,99/5) and perceived the app as useful (mean = 3,66/5).

**Discussion:**

Kalmer demonstrated potential to increase self-awareness, knowledge, and help-seeking attitudes. Areas for improvement include enhanced content personalization and user engagement. These findings support the feasibility of Kalmer as a complementary tool in NSSI treatment and lay groundwork for future clinical trials in public health contexts.

## 1 Introduction

Non-suicidal self-injury (NSSI) refers to the deliberate destruction of one's own body tissue without conscious suicidal intent (1, 2), encompassing methods such as cutting, burning and/or scratching the skin (3). NSSI represents a significant global public health challenge (4), as it imposes an economic burden on health systems and society at large. Specifically, NSSI places considerable weight on both the people who experience it and their loved ones.

It is especially concerning that the highest prevalence of the NSSI is among adolescents (5), because of its potential impact on emotional and cognitive development, including risks of suicidal behavior and psychological issues (5–7). As adolescence is a particularly vulnerable stage for developing mental health challenges, it is often a critical period for initiating NSSI, which can serve as a maladaptive coping mechanism to alleviate distress or express emotions (5). In fact, this phenomenon affects adolescents and young adults in both clinical and non-clinical contexts. It is estimated that about 17.2% of adolescents and 13.4% of young adults in community samples have experienced NSSI at some point in their lives (8, 9); while, in clinical samples, this prevalence is even higher, reaching up to 58% in adolescents (10). In addition, it is worth mentioning that rates are approximately twice as high in females compared to males in North America and Europe (7).

Moreover, NSSI is associated with adverse consequences, including difficulties in interpersonal relationships, academic performance, and daily functioning, and, most alarmingly, serves as a significant predictor of suicidal behavior (6, 7, 11). Recognized by the World Health Organization (WHO) as a major threat to adolescent health, NSSI has been classified in the latest edition of the *Diagnostic and Statistical Manual of Mental Disorders* as an independent disorder warranting further research. The emergence of this behavior during early adolescence has been linked to chronic psychosocial stress and experiences of rejection or victimization by peers (5, 12).

The primary function of non-suicidal self-injury (NSSI) is thought to involve the regulation of negative emotions and the restoration of emotional equilibrium (13). Evidence suggests that adolescents engaging in NSSI may become trapped in maladaptive coping cycles, wherein self-injurious behaviors, negative emotions, and dysfunctional thought patterns mutually reinforce each other over time, leading to heightened long-term risks (5). Distress tolerance (DT), defined as the capacity to endure negative internal states, has been identified as a transdiagnostic factor associated with various psychological disorders, including NSSI (6). Individuals with lower levels of DT tend to exhibit more intense emotional responses to stress and are more likely to adopt maladaptive coping strategies such as NSSI, whereas those with higher DT demonstrate healthier and more adaptive coping mechanisms (6). Emotional dysregulation, particularly heightened emotional states or “emotional cascades,” is considered central to the understanding of NSSI, as it increases the propensity for self-injurious behavior (6). Beyond emotional regulation, NSSI has been associated with additional functions, including self-punishment and the establishment of interpersonal boundaries (14).

Regarding its treatment, Dialectical Behavior Therapy (DBT) is considered the standard treatment for adolescents with NSSI (15). Therapies such as DBT and Cognitive Behavioral Therapy (CBT) focus on improving emotional regulation and can therefore be effective in addressing NSSI (16, 17). However, it is important to note that most of adolescents and young adults have difficulty seeking face-to-face help from mental health professionals (18). Considering the barriers of traditional therapy, less intensive treatment programs, specifically addressing NSSI, may be particularly appealing for young people (19, 20). For example, brief and self-administered online or mobile app-based interventions focused on NSSI alone could bridge the treatment gap by delivering real-time psychological assessments and interventions that integrate easily into patients' daily routines (19, 21).

Several mental health apps have shown effect sizes similar to conventional treatment standards across various conditions across different age groups, including adults (22), young adults (21), and adolescents (19, 20). Young people generally find digital interventions acceptable and show interest in using them. However, one common concern of digital interventions is the dropout rates. A review of Internet-based psychological treatments reported dropout rates ranging from 2 to 83%, with a weighted average of 31%, suggesting that adherence to digital intervention is very variable (23). A more recent study found that CBT delivered via the Internet (iCBT) had an average dropout of 16.3% (24). In short, these studies suggests that adherence to digital intervention is very variable and that multiple factors appear to influence dropout, including contextual, psychological or treatment-related variables (23, 24).

Within the specific field of NSSI, a recent review of web-based and mobile applications targeting self-injurious thoughts and behaviors found limited evidence of effectiveness in reducing NSSI (25), though some results appear promising. For example, the integration of DBT skills through a mobile app combined with face-to-face therapy has been linked to reducing urges for self-harm, decreasing NSSI frequency, lowering subjective distress, and improving self-efficacy and emotion regulation (26, 27). However, to date, only a few interventions have been developed that specifically target NSSI (28, 29).

Apart from treatment-seeking problems, these traditional therapies have other limitations, such as limited access due to scarce resources and a lack of trained professionals, a situation that has been exacerbated by the Covid-19 pandemic. Ecological Momentary Assessment (EMA) and Ecological Momentary Intervention (EMI) offer promising alternatives for overcoming traditional therapy barriers (30, 31).

EMA was developed as a way to gather data repeatedly within individuals' daily lives, prompting participants to answer questions about their current activities, emotions, or recent experiences at specific or random times (32). The widespread availability of smartphones (33) has further facilitated the use of EMA on a larger scale, providing an accessible and reliable method for gathering real-time data on individual experiences in different natural environments (32). In recent years, therapists and researchers have increasingly focused on enhancing the effectiveness of therapy by engaging patients within their daily environments (30). In this context, EMI leverages these principles, offering real-time prompts and interactions in response to EMA, extending traditional CBT into a digital format (32, 34). Emerging research highlights EMI's effectiveness in fostering self-awareness, establishing it as a powerful method for delivering immediate cognitive and behavioral interventions (21, 26, 32).

Due to the still scarce research on app-based therapy, it was decided to develop and validate a new app-based intervention program, based on EMA and EMI principles, for adolescents and young adults who engage in NSSI. The intervention includes CBT and DBT strategies. During the development phase, a usability study was carried out with a non-clinical population for ethical and technical reasons. As it was an initial version of the app, a verification of the functionality, usability, and quality was needed.

The primary aim of this usability pilot study was to evaluate the perceived usability and quality of the initial version of the specific app for NSSI, *Kalmer*, by a group of non-clinical participants with an age range from 16 to 25 years old. Usability, defined as the degree to which a product enables specific users to achieve their goals effectively, efficiently, and satisfactorily, is a critical factor in the success of health applications (35). Furthermore, the concept of usability encompasses important terms such as functionality, feasibility, acceptability and satisfaction; which will be tested in this study. Poor usability is a major factor which can lead to the underuse or discontinuation of health apps (35), making usability testing an essential aspect of technology assessment. The results of this preliminary evaluation will guide required changes and improvements in the app, and the optimized version will be tested in a clinical trial with adolescents and young adults who engage in NSSI behavior.

## 2 Method

### 2.1 Ethic and privacy

This study is included in a research project in which the LabPsiTec research group participates named *Design and analysis of the effectiveness of a brief mobile App-based intervention for Non-Suicidal Self-Injury: self-report, momentary and biological predictors of treatment outcomes*. This project has been approved by the ethics committee of the Jaume I University (UJI), (Ref, CEISH/25/2022), indicating that the project complies with the required ethical standards.

All procedures were approved by the UJI Human Research Ethics Committee (CEISH/25/2022). Regarding data security, the app conforms with required regulations, namely the General Data Protection Regulation (GDPR), Organic Law 3/2018 of 5 December and regulation 2016/679 of the European Parliament and of the Council (36). The data are stored on two servers connected locally at UJI, following the corresponding legal regulations. *Kalmer* app does not collect any personal data. The users are identified with an external anonymous ID provided by the researcher. This information is only known by the user and the lead researcher.

### 2.2 Participants

Participants were included in the study if they were in the range of 16–25 years old, they were willing to participate signing a consent form (inclusion criteria), and if they did not suffer from any emotional disorder (exclusion criteria). A non-clinical population was selected for ethical reasons and fast testing, as this was an initial version of *Kalmer*.

Forty-one adolescents and young adults were recruited through snowball technique. Of this number, only 34 downloaded the app. However, out of those 34, only 26 answered the final questionnaires on app usability and satisfaction. The drop out was 37% throughout the study.

The age range of the participants covered the target population of the study with an average age of 21.30 years (SD = 3.29). Most of the app users were women (77.8%) and 66.7% were university students. Also, 59.3% did not have a romantic partner and 55.6% did not have a job. As for mobile phones, only one participant did not have a personal mobile phone and the phone being an iOS or Android was equally distributed (48% for iOS and 52% for Android).

It is relevant to mention that from the total sample, 3 participants (12%) had incurred in NSSI behavior at some point in their life, while one of them had done it at least once in the last 12 months. None of the participants currently reported having a mental health problem, and none were receiving any treatment for a mental disorder.

### 2.3 Intervention design

The development was focused on designing a specific app-based intervention for NSSI, based on the principles of Cognitive Behavioral Therapy (CBT) and Dialectical Behavior Therapy (DBT). The focus of this app was to increase the number of strategies that users had to improve their well-being, distress tolerance, autonomy, ability to make choices, find meaning, feel more effective in social relationships, problem solving and decision making. For that to happen, an end-user-centered approach was followed for the development of *Kalmer*, based on participatory design and cooperative research.

In particular, the specific intervention was structured so that, depending on the participant's responses in the ecological momentary assessment (EMA), different ecological momentary interventions (EMIs) were offered. Therefore, depending on the needs of the participant at any given time, some tools were offered, either in video, image, or audio format. Specifically, the EMIs were grouped into five components: Distress Tolerance (DT), Emotion Regulation (ER), Mindfulness and self-compassion (M), Interpersonal Regulation (IR) and Problem-Focused Coping (PC).

As for the Ecological Momentary Assessments (EMAs), users could access the application at any time of the day to answer the 5 questions and, depending on the answers, receive an EMI appropriate to their state (On demand EMA). In addition, to ensure a response every day, they received a notification at 6:00 p.m. to respond to a scheduled EMA and a reminder of so at 7:00 p.m. Regarding the EMA, the questions that form part of it were slightly different depending if it was an “On Demand” EMA or the daily programmed EMA (Table 1). It was decided to add some questions regarding the use of social media (SM) to the programmed assessment, as its use is increasing in the target population and it has a correlation with NSSI behaviors (37, 38). EMAs were derived from widely used and psychometrically valid questionnaires for each component: 15-item Five Facet Mindfulness Questionnaire (**FFMQ-15)** (39, 40), 28-item Coping Orientation to Problems Experienced (**COPE-28**) (41, 42), Interpersonal Support Evaluation List-12 (**ISEL-12**) (43, 44), Difficulties in Emotion Regulation Scale (**DERS**) (45, 46) and the Inventory of Statements About Self-injury (**ISAS**) (47).

**Table 1:** Scheduled and on demand EMA questions.

Component	On Demand EMA	Scheduled EMA
Emotional Regulation (EM)	At this moment, to what extent do my emotions seem overwhelming to me?	At this moment, to what extent have my emotions seemed overwhelming?
Interpersonal Regulation (IR)	During the day today, to what extent did I spend more time on social networks than usual because I felt lonely and/or rejected?	At this moment, to what extent have I felt lonely and/or rejected?
Interpersonal Regulation II (SM)	–	During today, how many hours have I spent on social networks?
Interpersonal Regulation III (SM)	–	During the day today, to what extent did I spend more time on social networks than usual because I felt lonely and/or rejected?
Mindfulness (M)	At this moment, to what extent do I notice that my mind wanders off?	At this moment, to what extent did I notice that my mind wanders off?
Problem Coping (PC)	At this moment, to what extent do I feel able to cope with my problems?	At this moment, to what extent did I feel able to cope with my problems?
Distress Tolerance (DT)	At this moment, to what extent do I think about self-harm?	At this moment, to what extent have I thought about self-injury?

Regarding the Ecological Momentary Interventions (EMIs), one of the most important components for targeting the reduction of NSSI behaviors was Distress tolerance (DT), as it is related to the way individuals respond in stressful and intense emotional situations. As it was mentioned before, lower levels of DT are related to a higher likelihood of engaging in maladaptive coping strategies such as NSSI (6). Because of this, the EMIs related to DT are aimed to help the users in situations where the emotions are so intense and painful that they decide to engage in NSSI behavior. To this end, the strategies and tools offered are not aimed specifically to reduce the intensity of the emotion, but to help users not to fall into NSSI behaviors while the emotion returns to a controllable intensity. Another important component regarding NSSI behaviors is Emotion Regulation (ER), because of its relationship with how we cope with strong positive as well as negative emotions. Heightened emotions or “emotional cascades” increase the likelihood of self-injurious behaviors (6). In this case, the psychoeducation and strategies provided are meant to help users to manage their emotions by comprehending them and by having tools to reduce their intensity. The Mindfulness component is important as it provides users with the skills needed to focus on the present and understand how they really feel. With this knowledge, users can use the other strategies learned in a more efficient way. Finally, Interpersonal Regulation (IR) and Problem-Focused Coping (PC) are relevant components for adolescents and young people, as it is at this age when relationships with others become noteworthy and life problems and decisions start to appear and problems in these domains could appear as antecedents of NSSI (48). For that reason, offering strategies related to these areas can help them avoid abandonment and rejection feelings, which have been related to NSSI behaviors (5, 12).

Additionally, for the first week of app usage, 7 videos were created to introduce the use of the app and each component, in addition to increasing motivation and adherence. For the EMIs, a total of 32 videos were created to explain the different strategies and tools users could use to improve different aspects of their responses to daily problems and emotions. Moreover, 50 images and 42 infographics—similar to images, but with more content—were created to both explain more strategies and tools, to provide psychoeducation, and to remind the participants to use what they learned. Likewise, 8 audios were created, and 6 YouTube links were offered to give the participants more materials.

To determine which EMI should be offered according to the responses on the EMA, scores from 0 to 10 for each component were divided according to their relevance to the participant's physical and mental well-being. This division was established through expert judgement conducted by clinicians with extensive experience in the assessment and treatment of non-suicidal self-injury. The process was carried out collaboratively and by consensus among the experts, with the most important component being Distress Tolerance, as it is the most closely linked to the NSSI. If the user scored high in two components, which individually would lead to an EMI, the app prioritized whatever was most maladaptive in that moment.

This division was established through expert judgement conducted by a panel of clinicians with extensive experience in the assessment and treatment of non-suicidal self-injury. The process was carried out collaboratively and by consensus among the experts, with the most important component being Distress Tolerance, as it is the most closely linked to NSSI. If the user scored high in two components, which individually would lead to an EMI, the app prioritized whatever was most maladaptive in that moment.

**Figure 1:**
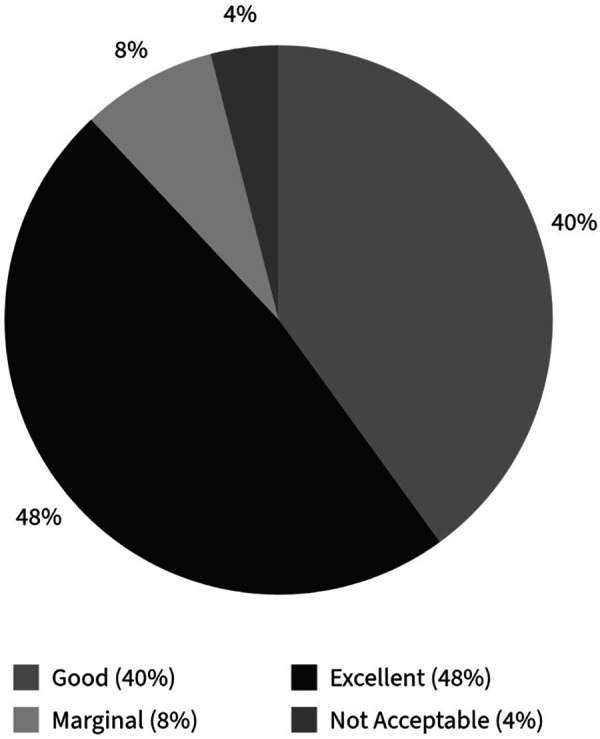
This is a screenshot of the main menu in *kalmer* app. It shows the displaying of the three principal options: “Answer now”, “Review my content” and “General Contents”.

In addition, the app main menu (Figure 1) included two more sections. Users could access the EMIs already received from the “Review My Contents” folder to review and practice them. Moreover, the app had another folder called “General Contents” were they had the phone number for the Suicidal Behavior Hotline, as well as some webs with information about self-injury and mental health.

### 2.4 Instruments

The sociodemographic data recollected was about gender, birth date, nationality, autonomous community of residence, study center (public, private or charter schools), whether or not they had a romantic relationship, marital status, number of children, level of education in progress or completed, whether or not they currently had a job, whether or not they had a mobile phone and which type of mobile operating system it had. Finally, questions about the exclusion criteria were made—whether they had a current diagnosis of mental disorder and whether they were receiving psychological treatment at that moment.

The Spanish adaptation of the *System Usability Scale* (*SUS)* (49, 50) was used to assess the app usability. The SUS scale is a widely used 10-item questionnaire scored on a 5-point Likert scale ranging from 1 (strong disagreement) to 5 (strong agreement). SUS scores range from 0 to 100, of which (51) suggest the most scientifically accepted interpretation of SUS.

The Spanish adaptation of the *User Version of the Mobile Application Rating Scale* (*uMARS*) (52, 53) provides an objective score of app quality and four scores from the same four objective quality subscales across 16 items rated on a 5-point Likert scale ranging from 1 (“inadequate”) to 5 (“excellent”), in addition to a “not applicable” option that was added to the response option. It also assesses the subjective quality with 4 items rated on a 5-point Likert scale with different options depending on the item. Besides the quality assessment, it also provides a perceived impact score from 6 items rated on a 5-point Likert scale ranging from 1 (“strongly disagree”) to 5 (“strongly agree”). The final score of each scale and subscales is calculated by averaging the items or subscale scores from each one.

### 2.5 Procedure

Participants between the age of 16 and 25 belonging to non-clinical population were recruited, covering the target population of the app: adolescents and young people. Each participant used the app for 2 weeks.

Recruitment was carried out by means of the “Snowball” method. Participants interested in the study contacted one of the researchers who sent them a link to Qualtrics survey, as well as a brief explanation of how the study works. The Qualtrics survey began with an explanation of the reason behind the study and its objectives. After signing the informed consent to participate, a series of questions were carried out to collect demographic data. In addition, it was also asked if they were currently receiving psychological treatment, since it was an exclusion criterion. It should be mentioned that to promote participation, among the participants who filled out the 3 questionnaires, 3 vouchers of €30 were raffled.

During the first week of the two-week study, the scheduled EMA referred participants to an introductory video of each component each day, while they had EMAs and EMIs available on demand (see Table 2).

**Table 2:** 1st week programmed videos.

Day	Video Content
1st day	Introduction to *Kalmer*
2nd day	Emotional Regulation
3rd day	Interpersonal Regulation
4th day	Problem Coping
5th day	Mindfulness
6th day	Distress Tolerance
7th day	Motivation for Change

During the second week, the app offered scheduled and on-demand EMAs and EMIs. Each time the user responds to the EMA, an EMI is offered if a cut-off point is exceeded. As mentioned above, if there are multiple components that appear to be affected, the EMI will be on the component of greater severity. Furthermore, as mentioned above, users could access the EMIs already received from the “Review My Contents” folder to review and practice them. They could also access another folder called “General Contents” where they had the phone number for the Suicidal Behavior Hotline, as well as some webs with information about self-injury and mental health. At the end of the second week, two more questionnaires were sent for the usability and quality assessment: the Spanish adaptation of the SUS (49, 50) and the Spanish adaptation of the uMARS (52, 53).

Even though participants only had to use the app for 2 weeks, the app was designed to offer a 9-week intervention. For this reason, participants could continue to use the app until the 9th week if they desired to do so.

### 2.6 Data analysis

The SPSS program version 23.00 (54) was used for data analysis, providing descriptive statistical analysis of sociodemographic data and quantitative results from the SUS and uMARS. Demographics were used to characterize the sample and exclude participants who did not meet the criteria. Finally, the SUS and uMARS scores as well as the data on the app usage were analyzed to explore the usability of the app.

SUS results were reported in the form of both total score and each 10-item means and standard deviations. For uMARS, the mean and standard deviation values for the overall objective quality score, the four objective quality subscales, and the total subjective quality score with its four related items were provided. Also, both mean and standard deviation values for the six items associated with perceived impact, along with the total score, were reported. Additionally, item-level means were examined to closely assess which aspects of the app required more improvement.

## 3 Results

### 3.1 Adherence to the App

Out of the 34 participants who downloaded the app, 26 (76%) completed the final questionnaire. However, the app usage of one of these 26 participants was not recorded due to an error with the entry code.

Adherence was calculated based on the number of EMAs responded to during the 15 days of app use. The average percentage of usage was 79.74% with a standard deviation (SD) of 33.23. More than 50% of the users completed all 15 scheduled EMAs (*n* = 16). Moreover, 8 participants used the app more days (from 4 to 16 extra days) after the 15-day study period.

Regarding non-scheduled EMAs, there was an average of 2.19 uses (SD = 3.01) during the two-week period, with a minimum of 0 and maximum of 14 uses.

### 3.2 Usability and quality of the App

#### 3.2.1 Usability assessment

**Figure 2:**
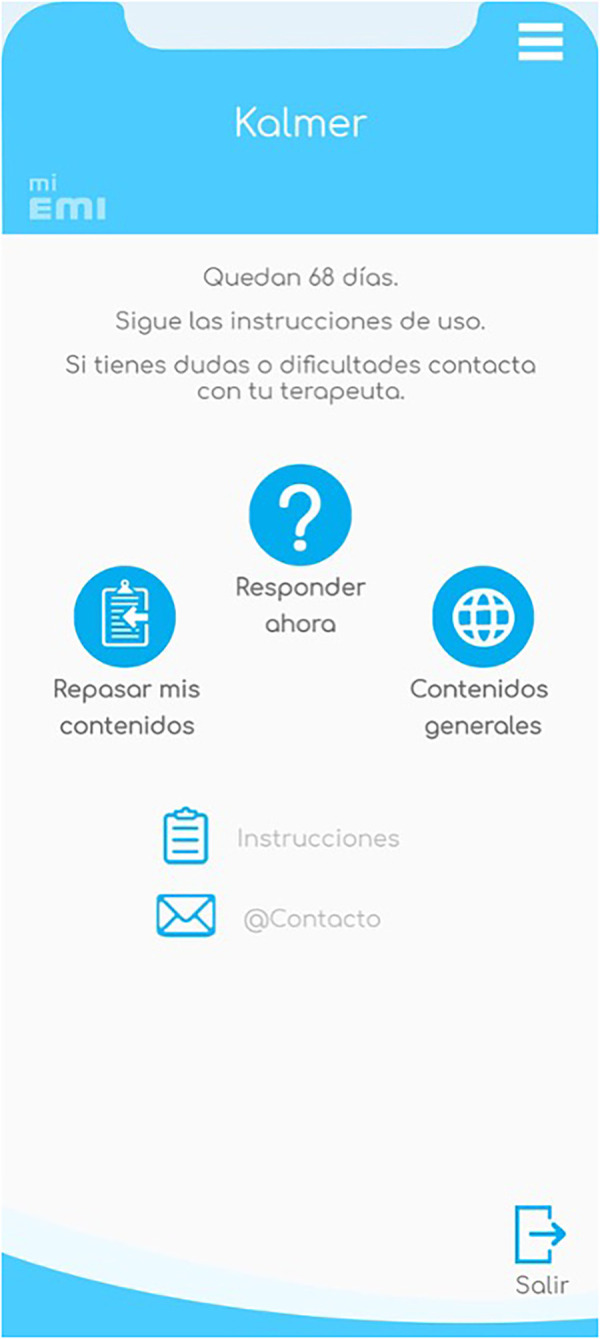
This figure shows the percentage of users that rated the App kalmer with different adjectives following the classification suggested by bangor et al. (51). Thus, total score in SUS between 0 and 52 were categorized as “Not Acceptable”, between 53 and 69 as “Marginal”, between 10 and 84 as “Good”, between 85 and 99 as “Excellent” and a total score of 100 as “Best Imaginable”.

The mean of SUS total score was 81.00 with a standard deviation (SD) of 12.99, being most of the score higher than 65 points. This result, according to the classification suggested by (51), means that the overall app usability could be rated as “Good”. Following the adjective rating classification proposed by these authors (Figure 2), 40% (*n* = 10) considered the overall app as “Good” and 48% as “Excellent” (*n* = 12). Only 8% (*n* = 2) considered the app had a “Marginal” usability and 4% (*n* = 1) as “Not Acceptable”.

Analyzing each item, the lowest score was for the negative items (good thing for the usability) that assess the complexity, difficulty of use and knowledge needed to use it (items 2, 4, 8 and 10). It is worth noting that the 6th item, also a negative one, related to the inconsistency of the app was the one with the highest mean score: 1.96 (SD = 0.89). Regarding the positive items, the ones with the highest score (good thing for the usability) were the ones that assess the ease of use (items 3 and 7). Items 5th and 9th, which assess the feature integration and confidence, had a quite high mean: 3.68 (SD = 0.95) and 3.76 (SD = 1.42) respectively. The lowest score for the positive items was the one which assessed the frequency of use the participants thought would have with the app, that is, the intention of use (M = 2.76; SD = 0,93). For more detail, the means and standard deviations of the item responses, along with the total usability score, are presented in Table 3.

**Table 3:** Descriptive statistics for SUS.

SUS items	M (SD)
1. I think I'd like to use this app frequently	2.76 (0.93)
2. I found the app unnecessarily complex	1.52 (0.82)
3. I thought the app was easy to use	4.60 (1.00)
4. I think I would need the help of technical staff to be able to use this app	1.12 (0.59)
5. I found the various features of this app to be well integrated	3.68 (0.95)
6. I thought there was too much inconsistency in this app	1.96 (0.89)
7. I imagine that most people could learn how to use this app very quickly	4.76 (0.66)
8. I found the app very difficult to use	1.28 (0.98)
9. I felt very confident using the app	3.76 (1.42)
10. I needed to learn a lot of things before I could start using this app	1.28 (0.98)
SUS total score	81.00 (12.99)

SUS, system usability scale; M, mean, SD, standard deviation; responses were scored on a 5-point Likert scale ranging from 1, strong disagreement to 5, strong agreement.

#### 3.2.2 Quality assessment

Starting with the Objective Quality (Table 4), the overall score was 3.88 (SD = 0.58), with 5 being the maximum rating possible. Going into detail on the objective quality subscales of uMARS, the highest rated subscales were the ones that assess the app functionality and the quality and quantity of information given. Participants considered the app worked correctly, was easy to use, and the information given was credible and visual. The item from the Information subscale with the lowest mean was the one referring to the ease of comprehension and conciseness of information given. On the other hand, the Entertainment subscale was the one with the lowest overall score, especially the items regarding the appearance of entertainment strategies and the possibility to personalize the app functions. However, the item referred to the appropriateness of the target population given the app design had a high mean score (M = 4.28; SD = 0.74). As for the last objective subscale, Aesthetics, users assessed the design as good enough, but the visual appeal could be improved. For further detail, means and standard deviations of objective quality subscales scores and items can be found in Table 5.

**Table 4:** Descriptive statistics for uMARS.

uMARS items	M (SD)	Range
App overall objective quality	3.88 (0.58)	2.03–4.60
Engagement	3.30 (0.65)	1.60–4.40
Functionality	4.46 (0.63)	2.50–5.00
Aesthetics	3.75 (0.70)	2.00–4.67
Information	4.02 (0.68)	2.00–5.00
Subjective quality	2.94 (0.69)	1.25–4.25
Would you recommend this app to people who might benefit from it?	3.28 (0.84)	1–5
How many times do you think you would use this app in the next 12 months if it was relevant to you?	3.56 (1.04)	1–5
Would you pay for this app?	1.80 (0.96)	1–4
What is your overall rating of the app?	3.12 (0.73)	1–4
Perceived impact	3.66 (0.97)	1–5
*Awareness*—This app is likely to increase awareness of the importance of addressing intense emotions more effectively	3.92 (1.00)	1–5
*Knowledge*—This app is likely to increase knowledge/understanding of how to deal with intense emotions more effectively	3.84 (1.07)	1–5
*Attitudes*—This app is likely to change attitudes toward addressing intense emotions more effectively	3.48 (1.12)	1–5
*Intention to change*—This app is likely to increase intentions/motivation to address intense emotions more effectively	3.44 (1.12)	1–5
*Help seeking*—Use of this app is likely to encourage further help to address intense emotions more effectively (if it's required)	3.88 (1.17)	1–5
*Behavior change*—Use of this app is likely increase addressing emotions more effectively	3.40 (0.96)	1–5

uMARS, User Version of the Mobile Application Rating Scale; M, mean; SD, standard deviation; Scale adapted from original English version in Stoyan*o*v et al. (53) and Spanish adaptation in Martin-Payo et al. (52). In this version, all objective quality items had the answer “not applicable”.

**Table 5:** Descriptive statistics for uMARS objective quality items.

uMARS items	M (SD)	Range
Engagement	3.30 (0.65)	1.60–4.40
*Entertainment*—Is the app fun/entertaining, does it use entertainment strategies to increase engagement (e.g., through games)?	2.63 (0.82)	1–4
*Interest*—Is the app interesting, does it present its content in an interesting way compared to other apps?	3.17 (1.05)	1–5
*Personalization*—Does it allow you to make adjustments or select preferences for configuration features (sound, content, notifications, etc.)?	2.90 (0.91)	1–5
*Interaction*—Do you allow user input, provide feedback, send notices?	3.23 (0.97)	1–5
*Target audience*—Is the content of the application (visual information, language, design) appropriate for the type of user it is aimed at?	4.28 (0.74)	3–5
Functionality	4.46 (0.63)	2.50–5.00
*Performance*—How fast/accurate are the features of the app (functions) and its components (buttons/menus) working?	4.28 (0.94)	1–5
*Ease of use*—Is it easy to learn how to use the app; Are the menu labels/icons and instructions clear?	4.72 (0.54)	3–5
*Navigation*—Is moving between screens logical/accurate/appropriate/uninterrupted; Are all the necessary screen links present?	4.44 (0.71)	3–5
*Gesture design*—Are interactions (keystrokes, keystrokes, swipes) consistent and intuitive across all components or screens?	4.40 (0.82)	2–5
Aesthetics	3.75 (0.10)	2.00–4.67
*Layout*—Is the layout and size of the buttons/icons/menus/contents of the screen adequate?	4.16 (0.90)	2–5
*Graphics*—How is the quality/resolution of the graphics used as buttons/icons/menus/content?	3.88 (0.83)	2–5
*Visual appeal*—Does the app look good?	3.20 (0.91)	1–5
Information	4.02 (0.68)	2.00–5.00
*Quality of information*—Is the content correct, well-written, and consistent with the objectives/themes of the application?	3.92 (0.76)	2–5
*Amount of information*—Is the information included in the app comprehensive and concise?	3.76 (0.78)	2–5
*Visual information*—Is the visual explanation of the concepts logical, clear and correct—through graphs/images/videos, etc.?	4.08 (0.81)	2–5
*Credibility*—Does the information contained in the app appear to come from a reliable source?	4.32 (0.95)	2–5

uMARS, User Version of the Mobile Application Rating Scale; M, mean; SD, standard deviation; Scale adapted from original English version in Stoyanov et al. (53) and Spanish adaptation in Martin-Payo et al. (52). In this version, all objective quality items had the answer “not applicable”.

Entering the Subjective Quality scale, the overall score was 2.94 (SD = 0.69). Here it is worth mentioning that every item had a mean score higher than 3 points, with 5 being the maximum rating possible; except for the item regarding the willingness to pay for using the app which had low mean score (M = 1.80; SD = 0.96). In the other 3 items (recommendation, use and general qualification) 88.5% of participants had a mean score higher than 3 points. Specifically, the item that assessed the use of the app in one year had the highest number of 5 points (19.2%); while no one assessed the general qualification of the app with the maximum score (57.7% with 3 points and 30.8% with 4 points).

Finally, the overall score for the Perceived Impact scale was 3.66 (SD = 0.97), with 5 being the maximum rating possible. The highest mean score was for the subscales referred to the app capability to increase awareness and knowledge about how to address adaptively intense emotions and to encourage asking for help when needed. The other subscales also had good mean scores. For further detail, means and standard deviations of objective quality subscales scores and subjective quality and perceived impact scores and items can be found in Table 4.

#### 3.2.3 Associations between app usage and usability and quality ratings

Spearman correlation analyses were conducted to explore the relationship between app usage (percentage of scheduled EMAs completed) and perceived usability and quality scores. No significant correlations were found between adherence and any of the usability or quality measures: SUS total score (*ρ* = .115, *p* = .591), Functionality (*ρ* = .329, *p* = .117), Aesthetics (*ρ* = 100, *p* = .641), Information (*ρ* = .204, *p* = .338), Objective Quality (*ρ* = .170, *p* = .427), Subjective Quality (*ρ* = −.005, *p* = .981), or Perceived Impact (*ρ* = −.119, *p* = .581). Regarding the subscales of the uMARS, strong and significant correlations were found among them and with the SUS total score. Notably, Aesthetics showed the strongest correlation with Objective Quality (*ρ* = .911, *p* < .001), and Functionality also correlated strongly with Objective Quality (*ρ* = .740, *p* < .001). Spearman correlation coefficients between the general variables are presented in Table 6.

**Table 6:** Spearman correlation matrix between app usage (adherence), usability (SUS) and quality measures (uMARS).

**Variable**	**1. SUS**	**2. Objective Quality**	**3. Subjective Quality**	**4. Perceived Impact**	**5. Adherence**	**M (SD)**
1. SUS	—					81.00 (12.99)
2. Objective Quality	.683**	—				3.88 (0.58)
3. Subjctive Quality	.622**	.638**	—			2.94 (0.69)
4. Percevied Impact	.585**	.607**	.498*	—		3.66 (0.97)
5. Adherence	.115	.170	-.005	-.119	—	79.74 (33.23)

Values in the lower triangle are Spearman's rho (*ρ*) coefficients. M, mean; SD, standard deviation. Adherence, percentage of scheduled EMAs completed.

* *p* < .05.

** *p* < .01.

Mann–Whitney *U*-tests were conducted to examine whether participants with higher adherence (≥80% of scheduled EMAs completed) differed from those with lower adherence (<80%) in their usability and quality ratings. No significant differences were found for any variable: SUS (*U* = 43.000, *p* = .748), Functionality (*U* = 25.500, *p* = .107), Aesthetics (*U* = 38.500, *p* = .515), Information (*U* = 39.500, *p* = .563), Objective Quality (*U* = 37.000, *p* = .455), Subjective Quality (*U* = 44.500, *p* = .829), or Perceived Impact (*U* = 45.000, *p* = .858).

Mann–Whitney *U*-tests were also conducted to explore potential gender differences. No significant differences were found between male (*n* = 5) and female (*n* = 20) participants on any measure: SUS (*U* = 33.500, *p* = .260), Functionality (*U* = 41.000, *p* = .530), Aesthetics (*U* = 35.500, *p* = .317), Information (*U* = 42.000, *p* = .581), Objective Quality (*U* = 41.000, *p* = .581), Subjective Quality (*U* = 35.000, *p* = .301), or Perceived Impact (*U* = 22.000, *p* = .056).

## 4 Discussion

The aim of this study was to assess the perceived usability and quality of the initial version of a specific app for NSSI by a target group of non-clinical participants. The results emphasize crucial areas and data that can support further research and practice in the mHealth sector, contributing to the improvement of this preliminary version of *Kalmer.*

Adherence results were good. Daily use of the app was maintained throughout the two weeks. This result is in line with the literature that finds good adherence during the first weeks of use (25). The inclusion of EMA reminders could help to encourage users to use the app every day (32), allowing them to receive immediate interventions more adapted to their needs and personalized to their changes in needs (30). The dropout rate was lower than what it is usually found in App-supported psychological interventions (23), but it was a little superior to what (24) found with iCBT interventions.

The adherence result is very important as a background to assessing app usability and acceptability, as it confirms that this data is based on regular use of the app. It seems that adolescents and young adults accept interventions based on apps and make use of them on a regular basis (19). As (20) observed, new psychological interventions based on apps can be a solution to the low demand for help from young people. This population seems to accept using apps (19) for psychological treatment and, as said, this can be of great help to improve the number of young people that seek help when needed (18). However, from the data collected, it can be observed the polarization of the adherence to the app: either the app is used a lot or hardly at all. In future research, it would be interesting to see if this small percentage of individuals who do not use the app is maintained in different contexts and populations and, if so, study the reasons behind their low engagement in order to tailor interventions to their needs.

As for usability and acceptability results (SUS), *Kalmer* was perceived as good by the users; with quite a large amount considering it as excellent. With respect to the items, users considered the app easy to understand and use, as well as safe for them. However, users also rated their intention to use the APP as low. This may be explained by the fact that the participants belonged to a non-clinical population with practically no-one incurring in NSSI behaviors, so an app designed to treat a pathological behavior is to be expected not to motivate them to use it. Also, the comments received from users were along these lines-they liked the app, but they did not find it useful for themselves, although they believed that people with psychological problems could benefit from it (26, 34). These aspects need more research to know what users' preferences regarding app designs are, as it may change depending on the target population and its context and needs.

The additional analyses conducted to explore patterns beyond descriptive statistics provided further insight into the app's usability profile. Strong and significant correlations were found between the SUS total score and the uMARS subscales, suggesting good convergent validity between the two instruments. The absence of significant correlations between adherence and usability or quality ratings suggests that how participants perceived the app was independent of how much they used it. This finding is relevant because it indicates that usability ratings were not artificially inflated by high engagement, nor penalized by low use—users who barely interacted with the app rated it similarly to those who used it extensively. A plausible interpretation is that usability and quality perceptions are formed relatively early in the interaction with the app and are not substantially shaped by cumulative exposure, at least over a two-week period. This interpretation, however, should be treated with caution given the small sample size and the limited statistical power of the analyses, particularly in the low-adherence and male subgroups. Regarding gender, no significant differences were found in any dimension, suggesting that the app was perceived similarly by male and female users. A non-significant trend was observed for Perceived Impact, with male participants showing higher ranks, which could be worth exploring in a larger and more gender-balanced sample in future studies.

Regarding *Kalmer* quality, it was rated as good by the users according to the uMARS results. On one side, about the objective quality results, the app seemed to work without significant problems in both Android and iOS mobile phones, which agrees with the comments received from the users. The errors regarding the no-registration of the data from the app and those regarding the repetition of some materials, as well as those regarding spelling mistakes, have been considered and the appropriate modifications are being made. The materials were reviewed to correct any kind of spelling mistake, and the technicians are checking the app programming to solve the other technical problems. Another strength of the app is the quality of its information, which was rated as correct, well-written, and consistent with the app's objectives. Participants also considered the content to be comprehensive and concise, the visual information—including graphs and images—to be logical and clear, and the overall information to be credible, appearing to come from a reliable source. It was also reported that the fact that the information and strategies were explained with different visual materials-videos, images, etc.—helped to understand the content better and to promote adherence in line with previous studies (20, 21).

On the other hand, Engagement was the subscale with the lowest overall score. Specifically, the users considered that the app was not very entertaining, which is understandable as it was designed as a psychological intervention. Also, the lower score in the interest item can be understood, like in the SUS, since participants did not deal with NSSI behaviors. However, users reported that some of the contents they received from the app were perceived as helpful for their usual activities, such as having more concentration while studying; or even with improving their mood or the way of dealing with life difficulties (19, 21). Also, the fact that different EMIs were shown in different contexts may help with the generalization of the strategies learned [i.e., (26)]. That may be as the strategies and tools are learned and reminded at various times and situations, the practice of them is more easily done in different moments, which could potentially lead to a faster generalization. However, this is just a hypothesis that needs more testing, as to what extent do EMAs and EMIs influence generalization.

It is noteworthy to mention that the item related to assessing if the app was appropriate to the target audience –adolescents and young adults- had a good overall score; meaning that the app is suitably designed for this population. Finally, the results from the last subscale, Aesthetics, show that the app design is well focused, but that some aspects could be improved, such as the visual impression the users have of it. Maybe making the app more visually appealing could help with adherence to both the use of the app and the use of the strategies learned through the app. As before, regarding Consistency and Integration, more research is needed to understand what this population expects from an app designed to treat psychological issues.

Regarding the Subjective Quality scale, the participants' overall rating of the app was generally good. Results also show that the participants would recommend the app to others who might benefit from it and that they will be willing to use the app for 1 year if they really needed it (20). However, the score that negatively affects the overall score of this scale is the item that refers to the willingness to pay for the use of the app. Most of the participants in this study said that they would not pay for it, something to be expected as they are from a non-clinical population and do not need the app for treatment. Nevertheless, this app is not being developed to be paid for in the future, but to be a tool to complement the treatment of people with NSSI behaviors in public health or educational settings (30).

Lastly, the Perceived Impact scale shows some good strengths of this app. The fact that the users perceive that the app helps them to be more aware of the importance of emotional regulation (6) and to be more inclined to ask for help if required is something really important for prevention and help seeking (18, 20). Whether this perceived increase in awareness and help-seeking attitudes could translate into meaningful clinical outcomes remains an open question that will need to be examined in future studies with clinical populations. Additionally, participants reported that the information given helped them to increase their knowledge about emotional regulation and how to deal with intense emotions in a more adaptative way (34). In fact, they reported higher perceived intentions to address intense emotions in a more adaptive way (6, 21, 26). To sum up, the Perceived Impact results suggest that users found the app content relevant and meaningful in their daily lives. However, it is important to note that these findings are based on measures obtained from a non-clinical sample and should not be interpreted as evidence of clinical effectiveness. Whether the app produces actual behavioral change in the target population—adolescents and young adults engaging in NSSI—remains to be determined in future clinical trials.

Finally, regarding the subjective feedback provided by users, it is being considered to improve the app. As mentioned before, there were some technical issues, such as videos repeating, that have been solved. To solve this error, the notification schedule has been modified so that it does not coincide between the first and subsequent weeks. Another change made was the introductory video, as during the study, there were some participants that did not understand quite well the difference between scheduled EMA and on-demand EMA. To address this confusion, the introductory video explaining the app's functions was updated to provide a more detailed explanation of this feature—considering the new changes in the notification hour. Also, the explanation of why the videos was generated with AI and do not appear “real people” has also been expanded—due to the need to protect the image and privacy of young people who may be feeling emotional discomfort. Lastly, some new material was created to add more audiovisual content at the request of users. One big change has been transforming infographics into videos, as they had too much text for small screens; as well as creating more videos guiding practical strategies, audios and images for Distress Tolerance and Mindfulness strategies and abilities-. In total, we created 10 more videos from infographics, 58 new images, 13 new audios, and 6 new video-exercises.

### 4.1 Limitations and improvement proposals

The study was done with non-clinical population even though the app has been designed for clinical population. This was an intentional methodological decision: testing the app in a non-clinical sample first allowed for the identification and correction of technical errors without exposing a vulnerable population to a potentially suboptimal version. Moreover, by using a non-clinical population, the presence of technical errors that could impede the proper functioning of the app does not harm the participants, as it is not a population that requires its use as a complement to psychological treatment.

It is acknowledged, however, that usability, perceived usefulness, and engagement are likely to differ between non-clinical users and individuals who actually engage in NSSI and represent the target population for the intervention. High usability scores in a non-clinical sample do not necessarily translate into sustained engagement or clinical relevance in the target population. The relatively low intention to use the app reported by participants illustrates this gap clearly, and conclusions regarding acceptability in the intended clinical population must therefore remain tentative. Importantly, it should be noted that long-term adherence patterns may also differ from those observed in this short usability study, and engagement may decline over time—a common challenge in digital mental health interventions that will need to be monitored in the upcoming clinical trial. Additionally, it is important to maintain a clear distinction between usability and quality—which are the focus of the present study—and clinical effectiveness, which was not assessed here and cannot be inferred from the current findings. The polarization in app usage observed in this study, whereby some users engaged extensively while others barely used the app, also warrants further investigation, as it may reflect meaningful individual differences in motivation or perceived relevance that are not captured by average scores. These issues will need to be addressed in the clinical study planned with the target population.

### 4.2 Improvement proposals and future research

In the future, it could be interesting to increase the app customization functions, such as allowing the users to decide the hour of the scheduled EMA notification and reminder. Another interesting improvement could be that, if an EMI has not reduced discomfort or has not been useful, another EMI is given-from the same component or different.

Regarding *Kalmer*, the next steps are validating the EMA questions and testing the app is in a real context with clinical population. This will be conducted through a pilot study in public hospitals in Catalonia, followed by a randomized controlled trial (RCT) aimed at assessing its effectiveness, implementation, and cost-effectiveness within the same public health system.

Moreover, upcoming projects should prioritize further enhancements to the app by integrating the improvement suggestions reported individually by participants. Also, input from professional psychologists and doctors will also be crucial in this process, as well as it will be important to explore the opinions and experiences of individuals who either do not respond to treatment or who drop out. This qualitative feedback could provide valuable insight which informs future changes of the app and its overall effectiveness in supporting users. By addressing these various dimensions, the aim is to create a more robust and user-centered application that meets the needs of its target population. Related to these needs, as the app will be used initially in Catalonia, a translated app from Spanish to Catalan could be interesting for giving a more personalized app.

## 5 Conclusion

In conclusion, this research has assessed the perceived usability and quality of the initial version of *Kalmer*, a specific app for NSSI, using a group of non-clinical participants from the target population-adolescents and young adults.

Adherence to the app during the study period was satisfactory, which provides a reasonable basis for interpreting the usability and acceptability results. Usability was generally perceived as good, and quality ratings were acceptable both objectively and subjectively. Additional analyses exploring the relationship between app usage and usability perceptions revealed no significant associations between adherence and any usability or quality measure, and no significant differences were found between high and low adherers or between male and female participants. These findings suggest that perceived usability and quality were consistent across different user profiles within this sample, though the limited sample size–particularly in the male and low-adherence subgroups–warrants caution in interpreting these results. Users also reported positive perceived impact in terms of self-awareness and emotional regulation knowledge. However, these results must be interpreted with caution: they are from a non-clinical sample, and they cannot be taken as indicators of clinical effectiveness. The primary goal of this study was to evaluate and refine the app from a technical and usability standpoint before proceeding to clinical testing. Whether Kalmer can produce meaningful outcomes for individuals who engage in NSSI will need to be determined through the planned clinical trial.

Finally, the various aspects identified as areas for improvement have played a significant role in refining the app. As a result, future efforts will be directed toward a rigorous clinical evaluation of the app with the target population, examining feasibility, acceptability, and—through a randomized controlled trial—its potential clinical impact when used in conjunction with standard therapeutic practices.

## Data Availability

The raw data supporting the conclusions of this article will be made available by the authors, without undue reservation.

## References

[1] Nock MK. Self-Injury. Annu Rev Clin Psychol. (2010) 6:339–63. 10.1146/annurev.clinpsy.121208.131258 20192787

[2] Vega D Sintes A Fernández M Puntí J Soler J Santamarina P. Review and update on non-suicidal self-injury: who, how and why? Actas Esp Psiquiatr. (2018) 46(4):146–55. 30079928

[3] Zetterqvist M. The DSM-5 diagnosis of nonsuicidal self-injury disorder: a review of the empirical literature. Child Adolesc Psychiatry Ment Health. (2015) 9(1):31. 10.1186/s13034-015-0062-7 26417387 PMC4584484

[4] Liu RT Scopelliti KM Pittman SK Zamora AS. Childhood maltreatment and non-suicidal self-injury: a systematic review and meta-analysis. Lancet Psychiatry. (2018) 5(1):51–64. 10.1016/S2215-0366(17)30469-8 29196062 PMC5743605

[5] De Luca L Pastore M Palladino BE Reime B Warth P Menesini E. The development of non-suicidal self-injury (NSSI) during adolescence: a systematic review and Bayesian meta-analysis. J Affect Disord. (2023) 339:648–59. 10.1016/j.jad.2023.07.091 37479039

[6] Akbari M Seydavi M Firoozabadi MA Babaeifard M. Distress tolerance and lifetime frequency of non-suicidal self-injury (NSSI): a systematic review and meta-analysis. Clin Psychol Psychother. (2024) 31(1):e2957. 10.1002/cpp.2957 38343352

[7] Moloney F Amini J Sinyor M Schaffer A Lanctôt KL Mitchell RHB. Sex differences in the global prevalence of nonsuicidal self-injury in adolescents: a meta-analysis. JAMA Netw Open. (2024) 7(6):e2415436. 10.1001/jamanetworkopen.2024.15436 38874927 PMC11179134

[8] Liu RT Walsh RFL Sheehan AE Cheek SM Sanzari CM. Prevalence and correlates of suicide and nonsuicidal self-injury in children: a systematic review and meta-analysis. JAMA Psychiatry. (2022) 79(7):718–26. 10.1001/jamapsychiatry.2022.1256 35612875 PMC9134039

[9] Swannell SV Martin GE Page A Hasking P St John NJ. Prevalence of nonsuicidal self-injury in nonclinical samples: systematic review, meta-analysis and meta-regression. Suicide Life Threat Behav. (2014) 44(3):273–303. 10.1111/sltb.12070 24422986

[10] Mendez I Sintes A Pascual JC Puntí J Lara A Briones-Buixassa L. Borderline personality traits mediate the relationship between low perceived social support and non-suicidal self-injury in a clinical sample of adolescents. J Affect Disord. (2022) 302:204–13. 10.1016/j.jad.2022.01.065 35038480

[11] Mars B Heron J Klonsky ED Moran P O’Connor RC Tilling K. Predictors of future suicide attempt among adolescents with suicidal thoughts or non-suicidal self-harm: a population-based birth cohort study. Lancet Psychiatry. (2019) 6(4):327–37. 10.1016/S2215-0366(19)30030-6 30879972 PMC6494973

[12] Brunner R Kaess M Parzer P Fischer G Carli V Hoven CW. Life-time prevalence and psychosocial correlates of adolescent direct self-injurious behavior: a comparative study of findings in 11 European countries. J Child Psychol Psychiatry. (2014) 55(4):337–48. 10.1111/jcpp.12166 24215434

[13] Taylor PJ Jomar K Dhingra K Forrester R Shahmalak U Dickson JM. A meta-analysis of the prevalence of different functions of non-suicidal self-injury. J Affect Disord. (2018) 227:759–69. 10.1016/j.jad.2017.11.073 29689691

[14] Klonsky ED. The functions of deliberate self-injury: a review of the evidence. Clin Psychol Rev. (2007) 27(2):226–39. 10.1016/j.cpr.2006.08.002 17014942

[15] Mehlum L Ramberg M Tørmoen AJ Haga E Diep LM Stanley BH. Dialectical behavior therapy compared with enhanced usual care for adolescents with repeated suicidal and self-harming behavior: outcomes over a one-year follow-up. J Am Acad Child Adolesc Psychiatry. (2016) 55(4):295–300. 10.1016/j.jaac.2016.01.005 27015720

[16] Kothgassner OD Robinson K Goreis A Ougrin D Plener PL. Does treatment method matter? A meta-analysis of the past 20 years of research on therapeutic interventions for self-harm and suicidal ideation in adolescents. Borderline Personal Disord Emot Dysregul. (2020) 7(1):9. 10.1186/s40479-020-00123-9 32426138 PMC7216729

[17] Ougrin D Tranah T Stahl D Moran P Asarnow JR. Therapeutic interventions for suicide attempts and self-harm in adolescents: systematic review and meta-analysis. J Am Acad Child Adolesc Psychiatry. (2015) 54(2):97–107.e2. 10.1016/j.jaac.2014.10.009 25617250

[18] Hasking P Rees CS Martin G Quigley J. What happens when you tell someone you self-injure? The effects of disclosing NSSI to adults and peers. BMC Public Health. (2015) 15(1):1039. 10.1186/s12889-015-2383-0 26453187 PMC4600263

[19] Bretón-López JM Mira Pastor A Castilla D García Palacios A Botella Arbona C. Revisión de aplicaciones de las tecnologías de la información y la comunicación en psicología clínica y de la salud en infancia y adolescencia. Rev Psicol Clínica Con Niños Adolesc. (2017) 4(3):11–6.

[20] Kaess M Koenig J Bauer S Moessner M Fischer-Waldschmidt G Mattern M. Self-injury: treatment, assessment, recovery (STAR): online intervention for adolescent non-suicidal self-injury—study protocol for a randomized controlled trial. Trials. (2019) 20(1):425. 10.1186/s13063-019-3501-6 31300065 PMC6626324

[21] Marciniak MA Shanahan L Myin-Germeys I Veer IM Yuen KSL Binder H. Imager—a mobile health mental imagery-based ecological momentary intervention targeting reward sensitivity: a randomized controlled trial. Appl Psychol Health Well-Being. (2024) 16(2):576–96. 10.1111/aphw.12505 37942875

[22] Simmonds-Buckley M Bennion MR Kellett S Millings A Hardy GE Moore RK. Acceptability and effectiveness of NHS-recommended e-therapies for depression, anxiety, and stress: meta-analysis. J Med Internet Res. (2020) 22(10):e17049. 10.2196/17049 33112238 PMC7657731

[23] Melville KM Casey LM Kavanagh DJ. Dropout from internet-based treatment for psychological disorders. Br J Clin Psychol. (2010) 49(4):455–71. 10.1348/014466509X472138 19799804

[24] Linardon J Messer M Reid R Bolger T Andersson G. Absolute and relative rates of treatment non-initiation, dropout, and attrition in internet-based and face-to-face cognitive-behavioral therapy: a meta-analysis of randomized controlled trials. Cogn Behav Ther. 4 de Agosto de (2025) 1–14. 10.1080/16506073.2025.2542364 40757987

[25] Arshad U Gauntlett J Husain N Chaudhry N Taylor PJ. A systematic review of the evidence supporting Mobile- and internet-based psychological interventions for self-harm. Suicide Life Threat Behav. (2020) 50(1):151–79. 10.1111/sltb.12583 31448847 PMC7027458

[26] Rizvi SL Dimeff LA Skutch J Carroll D Linehan MM. A pilot study of the DBT coach: an interactive Mobile phone application for individuals with borderline personality disorder and substance use disorder. Behav Ther. (2011) 42(4):589–600. 10.1016/j.beth.2011.01.003 22035988

[27] Stallard P Porter J Grist R. A smartphone app (BlueIce) for young people who self-harm: open phase 1 Pre-post trial. JMIR MHealth UHealth. (2018) 6(1):e8917. 10.2196/mhealth.8917 PMC5811647 29382632

[28] Julià A Jaén I García-Palacios A Pascual JC Sintes A Lara A. Delivering real-time support for self-injury: a systematic review on ecological momentary interventions. Internet Interv. (2025) 40:100826. 10.1016/j.invent.2025.100826 40342957 PMC12060517

[29] Turner BJ Austin SB Chapman AL. Treating nonsuicidal self-injury: a systematic review of psychological and pharmacological interventions. Can J Psychiatry. (2014) 59(11):576–85. 10.1177/070674371405901103 25565473 PMC4244876

[30] Kazdin AE. Interventions in everyday life to improve mental health and reduce symptoms of psychiatric disorders. Am Psychol. (2024) 79(2):185–209. 10.1037/amp0001158 37079813

[31] Shiffman S Stone AA Hufford MR. Ecological momentary assessment. Annu Rev Clin Psychol. (2008) 4:1–32. 10.1146/annurev.clinpsy.3.022806.091415 18509902

[32] Runyan JD Steinke EG. Virtues, ecological momentary assessment/intervention and smartphone technology. Front Psychol. (2015) 6:481. 10.3389/fpsyg.2015.00481 25999869 PMC4422021

[33] Ericsson. Mobility Report on the pulse of the networked society (2016).

[34] Heron KE Smyth JM. Ecological momentary interventions: incorporating mobile technology into psychosocial and health behaviour treatments. Br J Health Psychol. (2010) 15(1):1–39. 10.1348/135910709X466063 19646331 PMC2800172

[35] Torous J Wisniewski H Liu G Keshavan M. Mental health Mobile phone app usage, concerns, and benefits among psychiatric outpatients: comparative survey study. JMIR Ment Health. (2018) 5(4):e11715. 10.2196/11715 30446484 PMC6269625

[36] European Comission. General Data Protection Regulation. OJ L (2016). Available online at: http://data.europa.eu/eli/reg/2016/679/oj (Accessed January 20, 2026).

[37] Reddy N Rokito L Whitlock J. What is the link? The relationship between non-suicidal self-injury and social media. In: Information Brief Series, Cornell Research Program on Self-injury and Recovery. Ithaca, NY: Corne l University (2016). p. 1–4. https://www.selfinjury.bctr.cornell.edu/perch/resources

[38] Spínola LG Calaboiça C Carvalho IP. The use of social networking sites and its association with non-suicidal self-injury among children and adolescents: a systematic review. J Affect Disord Rep. (2024) 16:100781. 10.1016/j.jadr.2024.100781

[39] Baer RA Smith GT Hopkins J Krietemeyer J Toney L. Using self-report assessment methods to explore facets of mindfulness. Assessment. (2006) 13(1):27–45. 10.1177/1073191105283504 16443717

[40] Feliu-Soler A Pérez-Aranda A Luciano JV Demarzo M Mariño M Soler J. Psychometric properties of the 15-item five facet mindfulness questionnaire in a large sample of Spanish pilgrims. Mindfulness (N Y). (2021) 12(4):852–62. 10.1007/s12671-020-01549-6

[41] Carver CS. You want to measure coping but your protocol’ too long: consider the brief cope. Int J Behav Med. (1997) 4(1):92–100. 10.1207/s15327558ijbm0401_6 16250744

[42] Morán C Landero R González MT. COPE-28: un análisis psicométrico de la versión en español del brief COPE. Univ Psychol. (2009) 9(2):543–52. 10.11144/Javeriana.upsy9-2.capv

[43] Cohen S Mermelstein R Kamarck T Hoberman HM. Measuring the functional components of social support. En: Sarason IG Sarason BR, editores. Social Support: Theory, Research and Applications. Dordrecht: Springer Netherlands (1985). p. 73–94. Disponible en: 10.1007/978-94-009-5115-0_5 (citado 22 de enero de 2026).

[44] Merz EL Roesch SC Malcarne VL Penedo FJ Llabre MM Weitzman OB. Validation of interpersonal support evaluation list-12 (ISEL-12) scores among English- and Spanish-speaking hispanics/latinos from the HCHS/SOL sociocultural ancillary study. Psychol Assess. (2014) 26(2):384–94. 10.1037/a0035248 24320763 PMC4048059

[45] Gratz KL Roemer L. Multidimensional assessment of emotion regulation and dysregulation: development, factor structure, and initial validation of the difficulties in emotion regulation scale. J Psychopathol Behav Assess. (2008) 30(4):315. 10.1007/s10862-008-9102-4

[46] Hervás G Jódar R. Adaptación al castellano de la escala de dificultades en la regulación emocional. Clínica Salud. (2008) 19(2):139–56.

[47] Klonsky ED Glenn CR. Assessing the functions of non-suicidal self-injury: psychometric properties of the inventory of statements about self-injury (ISAS). J Psychopathol Behav Assess. (2009) 31(3):215–9. 10.1007/s10862-008-9107-z 29269992 PMC5736316

[48] Zhou J Zhang J Huang Y Zhao J Xiao Y Zhang S. Associations between coping styles, gender, their interaction and non-suicidal self-injury among middle school students in rural west China: a multicentre cross-sectional study. Front Psychiatry. (2022) 13:861917. 10.3389/fpsyt.2022.861917 36016979 PMC9395723

[49] Brook J. SUS: a quick and dirty usability scale. Usability Eval Ind. (1995) 189:4–7.

[50] Castilla D Jaen I Suso-Ribera C Garcia-Soriano G Zaragoza I Breton-Lopez J. Psychometric properties of the Spanish full and short forms of the system usability scale (SUS): detecting the effect of negatively worded items. Int J Human–Computer Interact. (2024) 40(15):4145–51. 10.1080/10447318.2023.2209840

[51] Bangor A Kortum PT Miller JT. An empirical evaluation of the system usability scale. Int J Human–Computer Interact. (2008) 24(6):574–94. 10.1080/10447310802205776

[52] Martin-Payo R Carrasco-Santos S Cuesta M Stoyan S Gonzalez-Mendez X Fernandez-Alvarez MdM. Spanish Adaptation and validation of the user version of the Mobile application rating scale (uMARS). J Am Med Inform Assoc. (2021) 28(12):2681–6. 10.1093/jamia/ocab216 34613400 PMC8633643

[53] Stoyanov SR Hides L Kavanagh DJ Wilson H. Development and validation of the user version of the Mobile application rating scale (uMARS). JMIR MHealth UHealth. (2016) 4(2):e5849. 10.2196/mhealth.5849 PMC4920963 27287964

[54] IBM Corp. IBM SPSS Statistics for Windows. Armok, NY: IBM Corp (2021).

